# Supplementary material for: Dynamics of spontaneous activity in random networks with multiple neuron subtypes and synaptic noise: Spontaneous activity in networks with synaptic noise
Source: J Comput Neurosci. 2018 Jun 19;45(1):1–28. doi: 10.1007/s10827-018-0688-6 (PMC6061197; doi:10.1007/s10827-018-0688-6)
Supplement: Supplementary file 1 — (PDF 1.45 MB) [file 10827_2018_688_MOESM1_ESM.pdf]

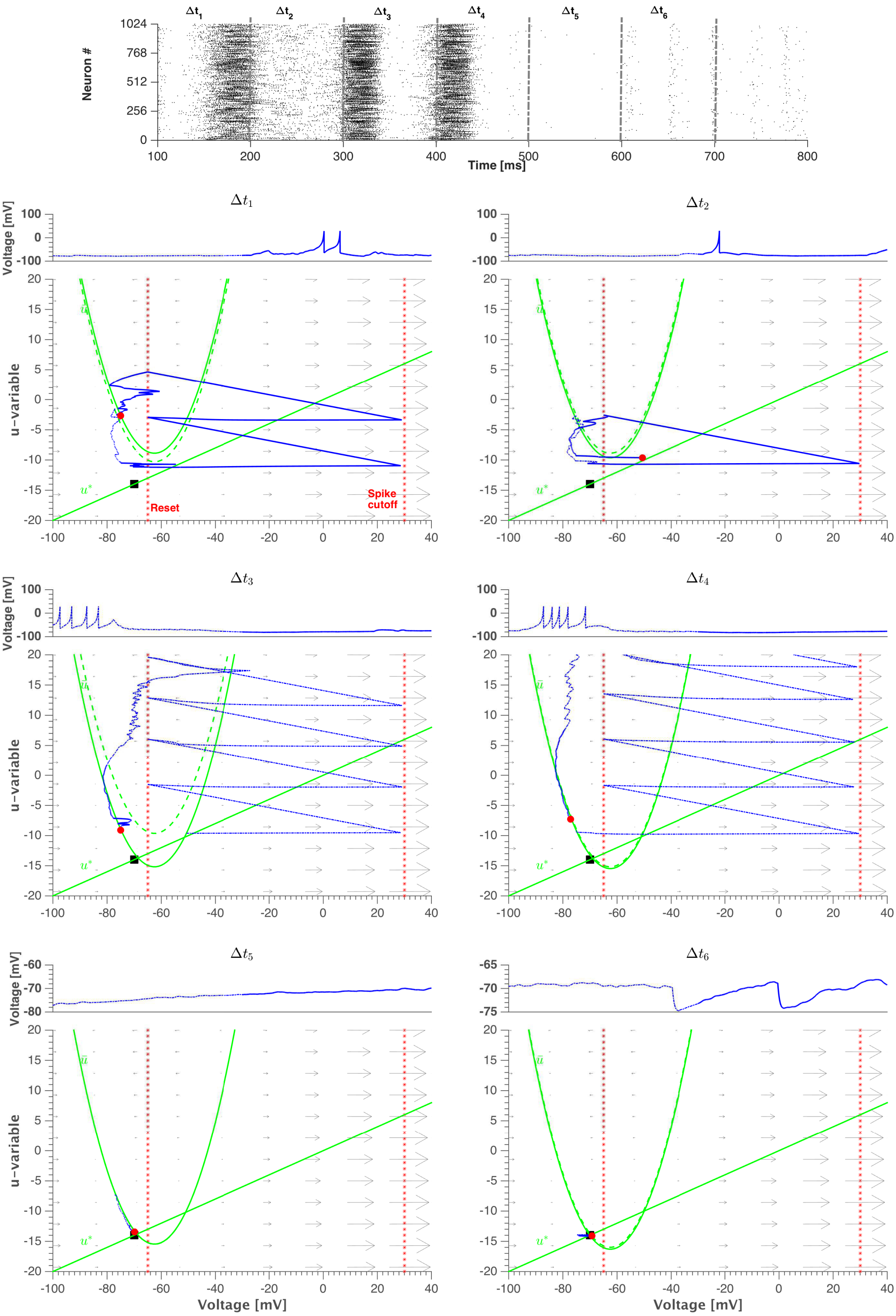

Fig. S1 Transient up-down oscillations in the weak synaptic noise setup. Network composition: 16%CH, 64%RS and 20%LTS neurons. Synaptic increments: $(g_{\rm ex}, g_{\rm in}) = (0.15, 1)$. Intensity of synaptic noise: $D = 2.5 \times 10^{-6}$. The network received a brief external stimulus from the beginning of simulation until $t = 80$ ms. After this the network was left to evolve freely until the end of the simulation. Upper panel: raster plot showing the firing activity in the network for $t \geq 100$ ms after the end of external input. The raster plot is divided into 6 time intervals Δt_i with duration 100 ms each. The network displays oscillatory behavior for a transient period followed by a quiescent state that lasts until the end of simulation. Lower panels: voltage series and dynamics on the phase plane of an arbitrary selected neuron in subsequent intervals Δt_i. Arrows indicate $(\dot{v}, \dot{u})$. Blue dashed line: the first 50 ms of evolution. Blue solid line: the last 50 ms of evolution. Red circle: location of the neuron at the end of the time interval. Black square: location of the state of rest with $v = v_{\rm rest}$ and $u = u_{\rm rest}$. Dotted red lines: reset value of voltage and spike cutoff. Green lines: Nullclines $\bar{u}$ and u^*, according to Eq (11). The location of the parabolic nullcline $\bar{u}$ is time-dependent; its position at the beginning (respectively, end) of Δt_i is shown with dashed (respectively, solid) green line.

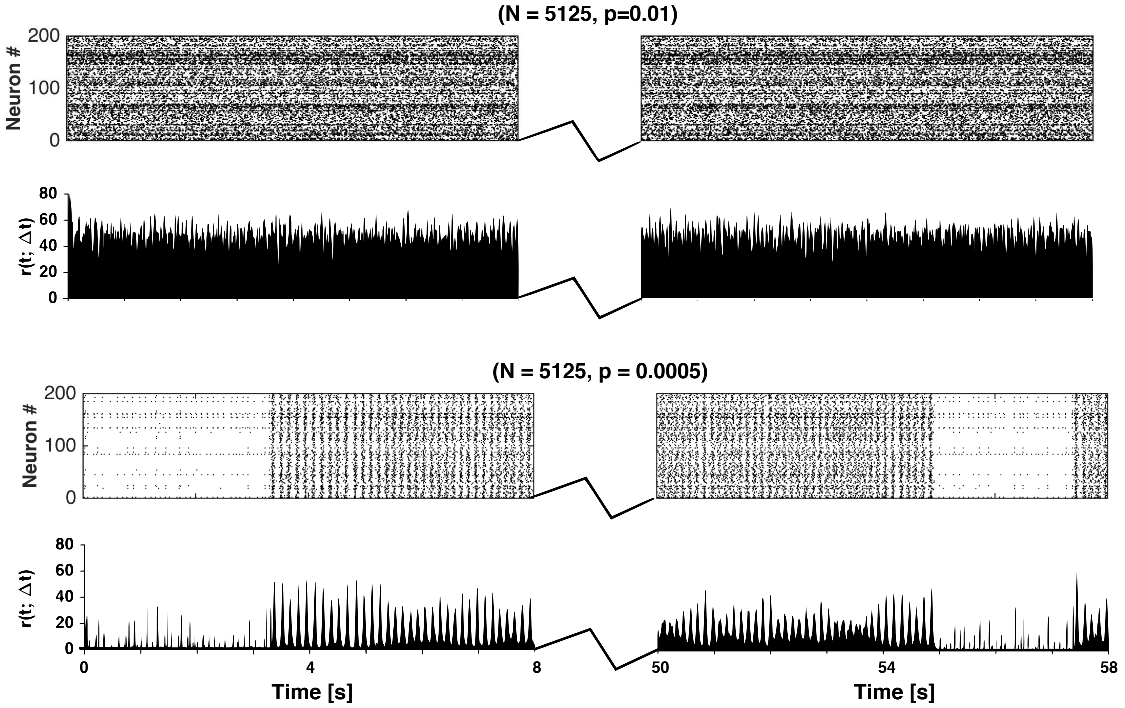

Fig. S2 Network size and connection probability dependence. In these simulations we used the same parameters as in Fig 8, except the number of neurons and connection probability. The upper panels correspond to a network with 5125 neurons and connection probability $p = 0.01$; the bottom ones concern the same number of neurons at $p = 0.0005$. Different from Fig 8 one can see that enlarging the network size makes its dynamics to work in an asynchronous constant activity mode. In contrast to that, by lowering the connection probability, the intermittent activity with both quiescent and active periods present is recovered.
